# Derangement of cardiac energy metabolism is acutely exacerbated during exercise in hypertrophic cardiomyopathy, independent of hypertrophy or late gadolinium burden

**DOI:** 10.1186/1532-429X-14-S1-O75

**Published:** 2012-02-01

**Authors:** Sairia Dass, Lowri E Cochlin, Joseph Suttie, Cameron Holloway, Christopher T Rodgers, Damian Tyler, Theodoros Karamitsos, Kieran Clarke, Hugh Watkins, Stefan Neubauer

**Affiliations:** 1 Deprtment of Cardiovascular Medicine, University of Oxford, Oxford, UK; 2 Deprtment of Physiology, Anatomy and Genetics, University of Oxford, Oxford, UK

## Summary

This work demonstrates that cardiac energetcics is further impaired during exercise in hypertrophic cardiomyopathy. This may be a possible reason for exercise related death in HCM.

## Background

In hypertrophic cardiomyopathy (HCM), sarcomere mutations increase the energy cost of contraction. Impaired resting cardiac energetics as measured by phosphocreatine/adenosine triphosphate (PCr/ATP) using 31Phosphorus MR Spectroscopy(31P MRS) has been documented in animal models and patients.

We hypothesize that: 1.Cardiac energetics are further impaired acutely during exercise in HCM, which does not occur in normals or athletes (physiological hypertrophy); 2. This impairment is not related to the degree of hypertrophy or late gadolinium enhancement (LGE) burden.

## Methods

Cardiac 31P MRS (3T) was performed in 35 HCM patients, 12 athletes and 20 normal controls (all age- and gender-matched) at rest and during 8 minutes of prone leg exercise with 2.5 kg weights attached to both legs. Cine and LGE images were also acquired.

## Results

Increases in rate pressure product with exercise were similar: normal 72±44%; HCM 73±38%; Athlete 75±47%.

**Figure 1:**
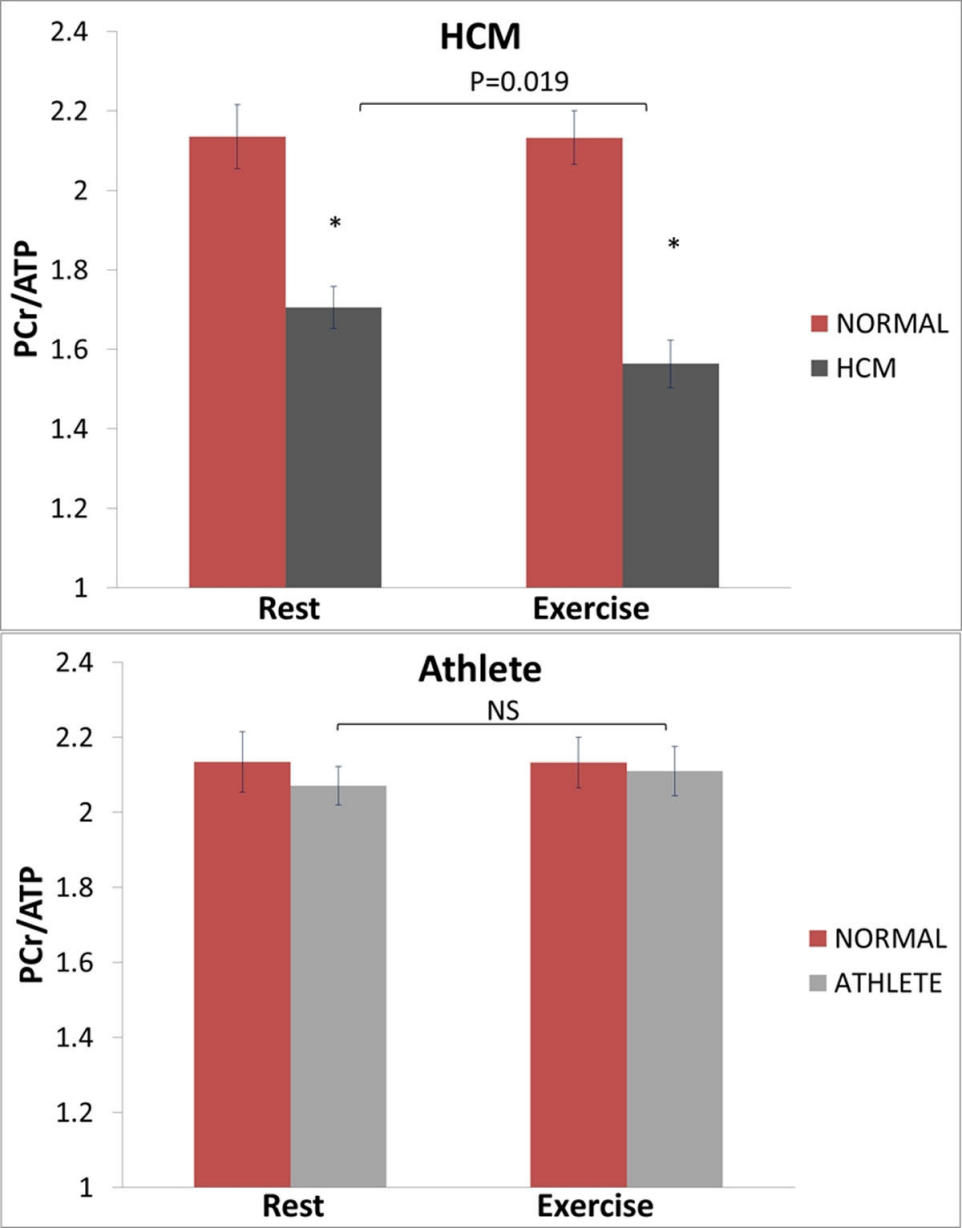
The effect of exercise on PCr/ATP in normal controls, HCM, and Athletes, showing a reduction in the HCM group, but no change in athletes or normal.*p<0.05 vs normal.

There was no difference in resting PCr/ATP between normals (2.14±0.36) and athletes (2.04±0.32, P=0.36). Resting PCr/ATP was significantly reduced in HCM, (1.71±0.35, P<0.01 compared to normal and athletes, figure 1).

**Figure 2:**
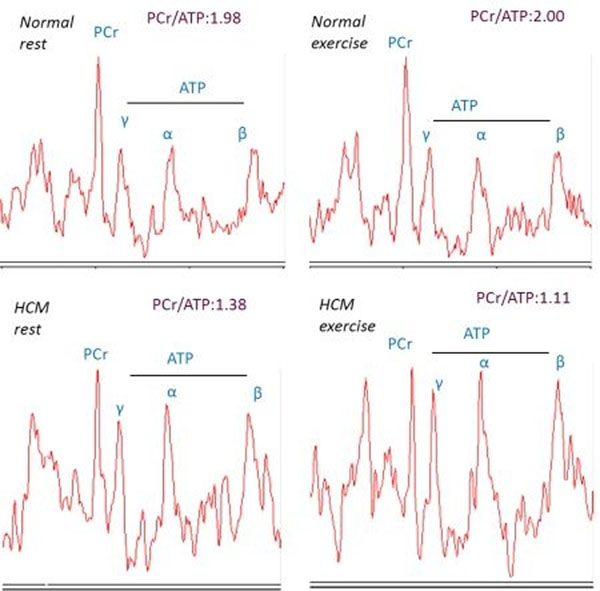
Examples of rest and exercise energetics in normal and HCM.

During exercise, there was a further reduction in PCr/ATP in HCM (1.56±0.31, P<0.05), but no significant change in normals (2.13±0.34, P=0.98), and athletes (2.09±0.50, P=0.63, figure 1). The change of PCr/ATP during exercise in HCM (-0.14±0.34) was significantly different (P<0.05) from the change in normals (+0.05±0.27).

LV mass index was higher in HCM (90±14g/m2) and athletes (92±19g/m2) compared to normal (65±10g/m2) P >0.05.There was no correlation between cardiac mass index and rest PCr/ATP ratios (HCM: R=0.01, P=NS; athletes: R=0.22 P=NS) or change in PCr/ATP with exercise.

In HCM, average wall thickness at voxel placement for PCr/ATP was 18±6mm (range 7.8-28.8 mm). This did not correlate with resting PCr/ ATP or change in energetics with exercise. Wall thicknesses were normal in the athlete group, 9±2mm.

Normals and athletes had no LGE. In HCM, the average LGE >2SD in the mid ventricular septum was 24±15%. LGE correlated weakly with resting PCr/ATP ratio, (R=- 0.35, P=0.04), and did not correlate with absolute exercise or change in PCr/ATP with exercise.

## Conclusions

During exercise, the pre-existing energetic deficit in HCM is further exacerbated and is not influenced by the degree of hypertrophy or scar burden. Acute derangement of energy-dependent ion homeostasis, triggering Ca++ overload and ventricular arrhythmias, may be a possible explanation for the high incidence of exercise-related death in HCM. Treatments that optimize energetics may be protective.

## Funding

This research was funded by the British heart foundation.

